# Thiol catalyzed formation of NO-ferroheme regulates canonical intravascular NO signaling

**DOI:** 10.21203/rs.3.rs-2402224/v1

**Published:** 2023-01-20

**Authors:** Anthony W. DeMartino, Laxman Poudel, Matthew R. Dent, Xiukai Chen, Qinzi Xu, Brendan S. Gladwin, Jesús Tejero, Swati Basu, Elmira Alipour, Yiyang Jiang, Jason J. Rose, Mark T. Gladwin, Daniel B. Kim-Shapiro

**Affiliations:** 1 Department of Medicine, University of Maryland School of Medicine, Baltimore, MD 21201, USA; 2 Department of Physics, Wake Forest University, Winston-Salem, NC 27109, USA; 3 Heart, Lung, Blood, and Vascular Medicine Institute, University of Pittsburgh, Pittsburgh, PA 15261, USA; 4 Division of Pulmonary, Allergy and Critical Care Medicine, University of Pittsburgh, Pittsburgh, PA 15261, USA; 5 Department of Bioengineering, University of Pittsburgh, Pittsburgh, PA 15260, USA; 6 Department of Pharmacology and Chemical Biology, University of Pittsburgh, Pittsburgh, Pennsylvania 15261, USA; 7 Translational Science Center, Wake Forest University, Winston-Salem, NC 27109, USA

## Abstract

Nitric oxide (NO) is an endogenously produced physiological signaling molecule that regulates blood flow and platelet activation. However, both the intracellular and intravascular diffusion of NO is severely limited by scavenging reactions with hemoglobin, myoglobin, and other hemoproteins, raising unanswered questions as to how free NO can signal in hemoprotein-rich environments, like blood and cardiomyocytes. We explored the hypothesis that NO could be stabilized as a ferrous heme-nitrosyl complex (Fe^2+^-NO, NO-ferroheme) either in solution within membranes or bound to albumin. Unexpectedly, we observed a rapid reaction of NO with free ferric heme (Fe^3+^) and a reduced thiol under physiological conditions to yield NO-ferroheme and a thiyl radical. This thiol-catalyzed reductive nitrosylation reaction occurs readily when the hemin is solubilized in lipophilic environments, such as red blood cell membranes, or bound to serum albumin. NO-ferroheme albumin is stable, even in the presence of excess oxyhemoglobin, and potently inhibits platelet activation. NO-ferroheme-albumin administered intravenously to mice dose-dependently vasodilates at low- to mid-nanomolar concentrations. In conclusion, we report the fastest rate of reductive nitrosylation observed to date to generate a NO-ferroheme molecule that resists oxidative inactivation, is soluble in cell membranes, and is transported intravascularly by albumin to promote potent vasodilation.

## Introduction

Heme (iron protoporphyrin IX) is an iron-containing prosthetic group, ubiquitous in biology. In blood, heme is found primarily in red blood cell hemoglobin, along with several plasma molecules including hemopexin, apolipoproteins, and albumin. It is also soluble in membrane lipids, is present in the erythrocyte membrane, and is actively transported from cell to cell via a number of processes still being elucidated.^1–5^ Nitric oxide (NO) is synthesized by nitric oxide synthase (NOS) and mediates canonical signaling via binding the reduced heme of soluble guanylate cyclase (sGC) with remarkably high selectivity and affinity (K_d_ = 5 × 10 ^−8^ – 4 × 10^−12^).^6^ Deoxygenating hemoglobin also produces NO and stimulates vasodilation via nitrite reduction, with observed NO formation as NO bound to the heme of hemoglobin (called iron nitrosyl hemoglobin) during physiological artery to vein deoxygenation.^7–10^ Additionally, red blood cells (RBCs) themselves are now known to contain active endothelial NOS (eNOS). The enzymatic activity of RBC eNOS generates NO and leads to the build-up of iron nitrosyl hemoglobin and contributes an independent role in blood pressure homeostasis.^11,12^ Antithetically, NO is also scavenged by hemoproteins such as oxygenated hemoglobin and myoglobin at almost diffusion-limited rates (~6–8 × 10^7^ M^−1^s^−1^ at 20°C),^13,14^ and reacts with deoxygenated hemoglobin and myoglobin at similarly high reaction rates to form iron-nitrosyl globins. Both reaction processes limit NO bioavailability to activate sGC.^13–15^

Although RBC hemoglobin has been shown to mediate NO signaling,^8,12,16,17^ one of the great mysteries in the NO field over the last twenty years is how NO, generated via nitrite reduction by RBC hemoglobin or RBC eNOS, escapes the red cell and can avoid the extremely fast and irreversible NO scavenging by hemoglobin to signal vasodilation. These scavenging reactions also present a conceptual challenge for intracellular NO signaling in cardiomyocytes and skeletal muscle myocytes, which contain high concentrations of myoglobin. While we know that NOS is active in these cells, modeling reaction kinetics and relative NO and myoglobin concentrations suggest myoglobin is present at sufficient concentrations to completely scavenge and inactivate NO in these cells.^18,19^ NO signaling in a hemoprotein-rich environment has been suggested to involve other species such as S-nitrosothiols^20–22^ or formation of other nitrogen oxides such as N_2_O_3_,^23–27^ as such species are relatively inert to autocapture and dioxygenation by hemoglobin and myoglobin. Our group and others have considered that a labile NO-bound ferrous heme or nitrosyl heme (NO-ferroheme) could serve as a possible intracellular signaling molecule and a way of protecting NO from autocapture and dioxygenation.^28,29^ Interestingly, early *in vitro* observations by Ignarro’s group indicate that nitrosyl heme can directly activate heme-free apo-sGC.^30^ Further, labile heme itself, without NO, is now appreciated as a signaling molecule.^31–35^ The details of intracellular heme trafficking are not completely understood, but molecules like glyceraldehyde-3-phosphate dehydrogenase (GAPDH) have been proposed to be involved, and such trafficking would play a role in signal amplification.^5,36,37^ With the discovery of ubiquitous heme transporters found on the membranes of many cells, including red blood cells,^1–3,38,39^ we hypothesized that a labile NO-ferroheme could transduce NO bioactivity in the vasculature. This hypothesis is supported by the observation that in humans, iron-nitrosylated hemoglobin – measured selectively by both chemiluminescence and by electron paramagnetic resonance (EPR) spectroscopy – forms in human blood endogenously during artery-to-vein physiological deoxygenation of blood, as nitrite is reduced to NO by deoxyhemoglobin and auto-captured as iron-nitrosyl-hemoglobin.^40,41^ The levels of this species increase when humans breath NO gas and during infusions of sodium nitrite.^8,29^ Importantly, we have previously observed unexpected artery-to-vein gradients in iron-nitrosylated hemoglobin during the inhalation of NO gas in humans, suggesting the possible delivery of NO from the erythrocyte, an observation that we could never explain considering the slow off-rate of NO per se from ferrous hemoglobin.^40^

Labile heme, taken to mean redox active heme weakly associated with proteins and thus readily exchangeable, is typically found in the ferric form due to its reduction potential, even in the presence of glutathione, though intercellular ferrous pools exist in high nanomolar concentrations.^1,4,42,43^ NO does reversibly bind to ferric heme but with generally much lower affinity than for ferrous heme.^44^ A nitrosyl-ferric heme with partial nitrosonium-ferrous heme character can then react with hydroxide to form nitrite and a ferrous heme, which rapidly binds any excess NO to form NO-ferroheme in a well-described process known as reductive nitrosylation.^45,46^ The reduction step is rate-limiting and very slow, with a half-life of several minutes depending on pH, and requires two molecule of NO to form one NO-ferroheme.^45^ This process is therefore an inefficient source of nitrosyl-ferrous heme *in vivo* as the reaction is slow^45^ and involves NO binding to low affinity ferric heme. The current studies demonstrate an alternative mechanism for rapid formation of a stable iron-nitrosylated heme that is first order in NO. Small, abundant biological thiols such as glutathione or cysteine facilitate an unexpected, physiologically viable, and fast reaction between NO and ferric heme, rapidly generating a nitrosyl-ferrous heme (NO-ferroheme) and a thiyl radical. Moreover, NO-ferroheme produced in this manner is taken up readily by red blood cell membranes and serum albumin, is stable in the presence of oxyhemoglobin, and exhibits potent vasodilatory responses *in vivo.*

## Results

### Reduced glutathione facilitates rapid formation of NO-ferroheme from NO and ferric heme in solution

**Figure 1:**
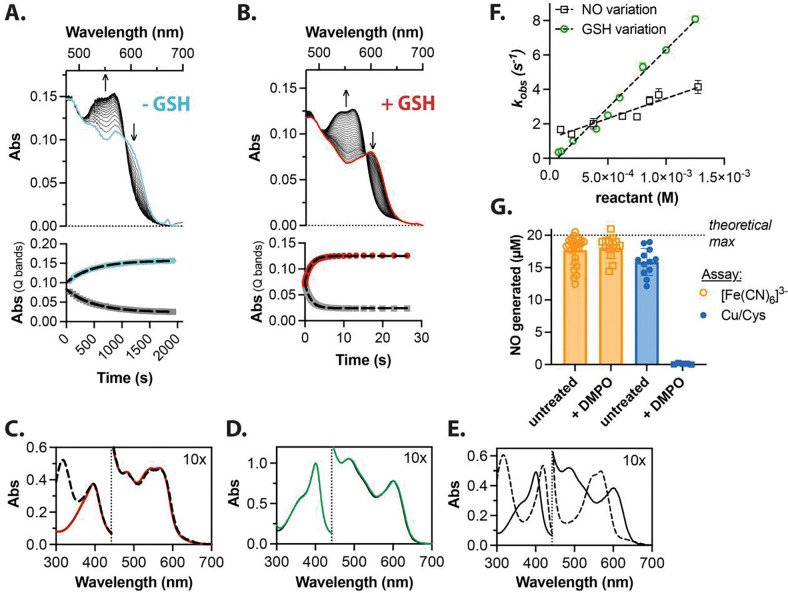
Basis spectra and reaction kinetics of reduced glutathione-assisted NO-ferroheme formation in anerobic MeOH:PBS buffer. **A)** Representative reductive nitrosylation of 12.5 μM ferric hemin by 1250 μM NO showing changes in the Q bands (*top*) and change over time at 570 and 611 nm (*bottom*). **B)** Glutathione catalyzed reductive nitrosylation of 12.5 μM ferric hemin with 125 μM GSH and 125 μM NO showing same regions as **A**. **C)** Addition of 250 μM NO to ferrous free heme – 12.5 μM hemin and 125 μM glutathione with ~100 μM sodium dithionite – results in NO-ferroheme observed in **A** and **B** (red line), with the characteristic dithionite peak at 315 nm (hashed line). **D)** The spectra of 25 μM ferric heme (black line) and 25 μM ferric heme with 250 μM glutathione (green line) show no appreciable difference, nor signs of reduction under these conditions. **E)** 12.5 μM ferric heme and 125 μM GSH (solid line) was reacted with ~100 μM sodium dithionite, generating the ferrous spectra (hashed line). **F)** Observed pseudo first order rate constants vs concentration of either GSH variation (green open circle, NO concentration held at 250 μM) or NO variation (black open squares, GSH concentration held at 250 μM) resulting in slopes (second order rate constants) of 7000 and 2300 M^−1^s^−1^, respectively. **G)** Stoichiometry of the thiol catalyzed reaction in MeOH:PBS buffer determined by quantification of NO in a chemiluminescent nitric oxide analyzer. Injection of aliquots of 20 μM solutions based on heme into ferricyanide solution (orange circles) results in production of 17.7 ± 2.2 μM NO; injection of the same solution into Cu/Cys (2C assay) generates 16.1 ± 2.1 μM NO, indicated the presence of roughly 1 equivalent of NO-ferroheme to 1 equivalent of GSNO (‘untreated’ bars). If instead the reaction is pretreated with 50 mM DMPO before adding NO to initiate the NO-ferroheme formation, 18.1 ± 1.7 μM of NO was detected from the ferricyanide assay and 0.1 ± 0.09 μM of NO were detected from the 2C assay (‘+ DMPO’ bars), indicating formation of a thiyl radical which is consumed by DMPO before formation of GSNO. Each point represents an injection into the analyzer purge vessel, of at least four separate preparations. All kinetic reactions were done at 23°C. See main body of the text for more details.

To explore the reaction between ferric heme, reduced glutathione (GSH), and NO, we used a 5:1 mixture of methanol to phosphate buffered saline (PBS, pH 7.4) – herein referred to as the MeOH:PBS buffer – to fully solubilize free heme at concentrations amenable to UV-visible spectroscopic characterization. Addition of 1250 μM NO to a mixture containing 12.5 μM ferric heme under anerobic conditions at 23 °C results in slow (*t*_1/2_ = 340 s) formation of NO-ferroheme (Figure 1A) with Q-band peaks at 543 and 568 nm, consistent with classic reductive nitrosylation reaction of two molecules of NO with ferric heme, yielding nitrite^45^ followed by rapid NO binding to the resulting ferroheme (Eqs. 1 and 2– where PPIX is protoporphyrin IX).

(1) $$[\text{NO}-{\text{Fe}}^{3+}(\text{PPIX}){↔}^{\;+}\text{NO}-{\text{Fe}}^{2+}(\;\text{PPIX)]}+{\text{OH}}^{-}\to {{\text{NO}}_{2}}^{-}+{\text{Fe}}^{2+}(\;\text{PPIX)}\;)+{\text{H}}^{+}$$

(2) $${\text{Fe}}^{2+}(\text{PPIX})+\text{NO}\to \text{NO}-{\text{Fe}}^{2+}(\text{PPIX)}$$

Unexpectedly, addition of 125 μM glutathione with 12.5 μM ferric heme and only 125 μM NO results in rapid formation a product with the same spectroscopic signature, indicating NO-ferroheme formation (Figure 1B, note markedly different time scale from 1A). This spectroscopic signature (Figure 1C, *red line*) is also recapitulated in the absence of glutathione with the addition of 20-fold excess NO to 30 μM heme reduced with excess sodium dithionite (Figure 1C, *hashed line*), though the glutathione-facilitated product lacks the characteristic dithionite absorbance at 315 nm. Addition of 300 μM GSH to 30 μM ferric heme in the absence of NO (Figure 1D) resulted in no observable spectral changes, nor is the unliganded ferrous form observed (Figure 1E, *hashed line*), suggesting the glutathione does not coordinate or reduce heme under these conditions, respectively.

Varying the concentrations of NO or reduced glutathione under pseudo first order conditions allows for determination of the dependence of this reaction on either GSH or NO, yielding calculated second order rate constants of 7000 M^−1^s^−1^ and 2300 M^−1^s^−1^, respectively (Figure 1F). All spectra showed isosbestic points at 503 and 594 nm (e.g., Figure 1B, *top*), and traces (e.g., Figure 1B, *bottom*) fit well to single exponential kinetic analyses, together indicating a two-species rate limiting reaction without significant formation of intermediates to generate NO-ferroheme. The speed of this reaction was unexpected and to our knowledge is the fastest rate of reductive nitrosylation observed to date for a heme protein.

As discussed further below, the identity and stoichiometry of the reaction products supports a mechanism involving thiol-catalyzed reductive nitrosylation of ferric heme to form NO-ferroheme and a thiyl radical, which subsequently reacts with excess NO to form secondary S-nitrosoglutathione (GSNO). These nitrosyl species, NO-ferroheme and GSNO, were identified and quantified by reductive chemiluminescence with NO detection in the chemiluminescent nitric oxide analyzer (Figure 1G, ‘*Untreated’ bars*). Specific detection of NO derived from NO-ferroheme (no NO release from GSNO) was achieved through chemical oxidation with potassium ferricyanide, which oxidizes ferrous nitrosyl species and liberates NO without degrading GSNO.^47^ Aliquots of an anaerobic reaction mixture containing 20 μM ferric heme, 200 μM GSH, and 40 μM NO in MeOH:PBS buffer after 10 minutes of reaction time were added to the nitric oxide analyzer purge vessel containing 10 mM potassium ferricyanide (**Supplementary Figure 1A**). Each injection corresponded to roughly 1 equivalent of NO (17.7 ± 2.2 μM) per equivalent of generated NO-ferroheme (**Supplementary Figure 1C**). Specific detection of NO derived from GSNO (no NO release from NO-ferroheme) was achieved using the well-described copper/cysteine (Cu/Cys, 2C) method for S-nitrosothiol detection (**Supplementary Figure 1B**),^48^ as NO-ferroheme itself does not degrade spectroscopically in the presence of Cu/Cys (data not shown). In the presence of the Cu/Cys mixture, the reaction mixture containing 20 μM ferric heme, 200 μM GSH, and 40 μM NO in MeOH:PBS buffer yielded 15.9 ± 2.1 μM of NO per injection (**Supplementary Figure 1D**). We anticipate a slightly sub-stoichiometric yield of GSNO-derived NO due to oxidized glutathione (GSSG) formation and/or GSNO degradation, which occurs in the presence of the excess reduced thiol.^49^ Taken together, these data suggest the formation of NO-ferroheme and GSNO in roughly a 1:1 stoichiometry.

A few possible reaction mechanisms account for the formation of NO-ferroheme and GSNO in a 1:1 stoichiometry. First, NO-ferroheme and GSNO formation may be the result of thiol nucleophilic attack of a transient ferrous heme-nitrosonium (Eq. 3) followed by rapid binding of a second equivalent of NO to the ferrous heme (Eq. 2), as observed in traditional reductive nitrosylation (*i.e.,* base-dependent and nitrite-catalyzed reductive nitrosylation).^27,45^

(3) $$\left[\text{NO}-{\text{Fe}}^{3+}(\;\text{PPIX}\;){↔}^{\;+}\text{NO}-{\text{Fe}}^{2+}(\;\text{PPIX)}\right]+\text{GSH}\to \text{GSNO}+{\text{Fe}}^{2+}(\text{PPIX})+{\text{H}}^{+}$$

Second, generally, glutathione may undergo a one-electron oxidation to reduce a ferric heme nitrosyl to ferrous heme nitrosyl, followed by rapid reaction thiyl radical with an equivalent of NO (Eqs. 4 and 5), though this electron transfer may occur via an inner sphere or outer sphere electron transfer mechanism (*vide infra*). In the case of inner sphere electron transfer, the thiol may initially bind free ferriheme or pre-formed NO-ferriheme, though this order of addition cannot readily be determined from the reaction kinetics or stoichiometry.

(4) $$\text{GSH}+\text{NO}-{\text{Fe}}^{3+}(\text{PPIX})\to {\text{GS}}^{\cdot }(thiyl\;radical)+\text{NO}-{\text{Fe}}^{2+}(\text{PPIX})+{\text{H}}^{+}$$

(5) $${\text{GS}}^{\cdot }+\text{NO}\to \text{GSNO}$$

These two mechanisms – traditional reductive nitrosylation vs. thiol-catalyzed reductive nitrosylation – yield the same products, though not necessarily in equivalent amounts. The traditional reductive nitrosylation mechanism does not yield a radical intermediate and, as it is base driven, should also result in a considerable amount of nitrite and a sub-stoichiometric amount of GSNO. Moreover, as described above, the observed reaction kinetics of NO-ferroheme formation are much faster than those observed for traditional reductive nitrosylation, suggesting that a thiyl radical-generating mechanism is operative.

To determine categorically that the thiyl-generating mechanism predominates in this system, we directly probed radical formation by reacting ferric heme, GSH, and NO in the presence of the radical trap 5,5-dimethyl-1-pyrroline-N-oxide (DMPO). It is worth noting that while NO itself is a radical, it does not react with DMPO.^50^ We quantified the formation of NO-ferroheme and GSNO under these conditions from 20 μM hemin using the chemiluminescence NO analyzer as described above. In MeOH:PBS buffer, DMPO completely abrogated the formation of GSNO (0.1 ± 0.09 μM, **Supplementary Figure 1E**) while preserving NO-ferroheme formation (18.1 ± 1.7 μM), providing strong evidence for the thiyl radical hypothesis and thiol-catalyzed reductive nitrosylation (Figure 1G, *‘+ DMPO’ bars*).

### NO-ferroheme formation in hemoglobin-depleted red blood cell membrane white ghosts

**Figure 2:**
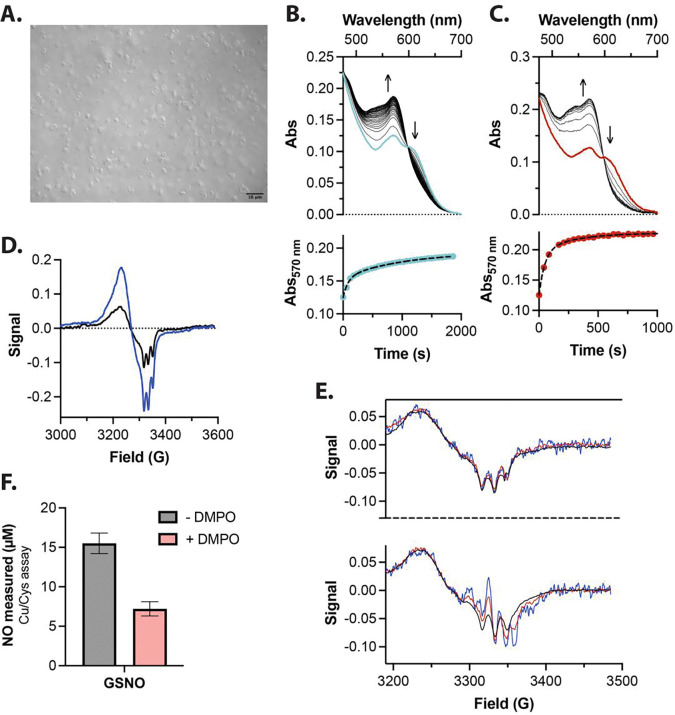
Reduced glutathione accelerates the reaction of NO and ferric heme in suspended RBC membranes (‘ghosts’) under anaerobic conditions. **A)** Differential interface contrast image of red cell ghost preparation (10% by volume) used to mimic an actual biological space where this chemistry can occur. Bar represents 16 μm. **B)** Adding 50 μM NO to 25 μM ferric heme solubilized in the ghosts results in reductive nitrosylation in the membranes. The time course follows the formation of NO-ferroheme at 570 nm. **C)** The same reaction as **B**, except with 250 μM added GSH in solution, resulting in a greatly increased rate NO-ferroheme formation, despite the lipophilicity of the membranes and the relative GSH solubility. **D)** EPR measurements of the final products could be made using the ghost’s system: without GSH (black line, 5.2 μM NO-ferroheme) and with GSH added (blue line, 13.5 μM NO-ferroheme). **E)** *Top,* 25 μM ferric heme, 50 μM GSH, and 50 μM NO were reacted in the RBC membrane ghost system and EPR spectra recorded at three different powers: 0.1 (black line), 1 (red line), and 10 mW (blue line). The spectra overlap after normalization (dividing raw intensity by the square root of the power). *Bottom,* This same reaction was carried out in the presence of 50 mM DMPO and EPR spectra recorded at the same three powers. Here, however, an organic radical saturates at higher powers, demonstrating formation of the DMPO-glutathionyl radical. EPR spectra were collected at 110 K. **F**) Chemiluminescent measurement of GSNO using the 2C assay of the reactions in Figure 1G show blunting of S-nitrosothiol formation in the presence of DMPO. All reactions were carried out under anaerobic conditions.

Hemin (ferric heme) is hydrophobic and must be solubilized in organic solvents like methanol. *In vivo,* heme is solubilized in cell lipid bilayers and is particularly abundant in the erythrocyte membrane, though this has predominantly been investigated in the context of free heme toxicity and lipid peroxidation.^51,52^ To demonstrate that thiol catalyzed reductive nitrosylation occurs in a physiologically relevant context, we employed suspended red blood cell “white ghost” membranes, prepared as previously described,^53^ to solubilize heme (Figure 2A).^54^ NO-ferroheme formation in membrane suspensions was measured using a UV-Vis spectrophotometer with an integrating sphere detector. Consistent with observations in MeOH:PBS solution, a slow reaction characteristic of reductive nitrosylation was observed in ghost membrane suspensions bearing ferric heme and NO in the absence of thiol (Figure 2B). However, the reaction rate of NO-ferroheme formation was greatly enhanced by the addition of a stoichiometric excess of GSH to ferric heme, resulting in formation of a product spectroscopically analogous to that observed in the MeOH:PBS system (Figure 2C, here 250 μM GSH). Importantly, the use of RBC membrane ghosts enabled characterization of the NO-ferroheme product by low temperature electron paramagnetic resonance (EPR) spectroscopy. Both reactions (in the presence and absence glutathione) give rise to a rhombic EPR spectrum characteristic of a five-coordinate NO-ferroheme species with *g*-values at 2.081, 2.054, and 2.012 for g_max_ , g_mid_ , and g_min_ , respectively, and a three-line hyperfine splitting centered on g_min_ (^NO^A = 47 MHz,, Figure 2D).^55,56^

As described earlier, DMPO reacts with a glutathionyl radical to form a stable adduct that is detectable by EPR spectroscopy. To directly probe formation of the glutathionyl radical under physiologically relevant conditions, we generated NO-ferroheme solubilized in red blood cell ghosts in the presence and absence of excess DMPO. While the heme nitrosyl EPR signal intensity remains proportional to the square root of the microwave power, the signal derived from the DMPO-glutathionyl adduct saturates at higher power.^57^ Addition of ferric heme, excess NO, and excess glutathione in membranes without DMPO results in the typical ferrous nitrosyl spectrum overlapping at all three power levels after normalization to the square root of these powers (Figure 2E, *top*). In the same reaction with the addition of 50 mM DMPO, a signal characteristic of the DMPO-glutathionyl spin adduct, centered at 3350 G, saturates as power increases (Figure 2E, *bottom*). We quantified GSNO quantification using Cu/Cys based reductive chemiluminescence in the NO analyzer with and without DMPO in this aqueous RBC membrane ghosts system; GSNO production was significantly inhibited by DMPO (Figure 2F).

### Reaction of NO, heme, and glutathione with albumin forms albumin-bound nitrosyl heme under physiological conditions

**Figure 3:**
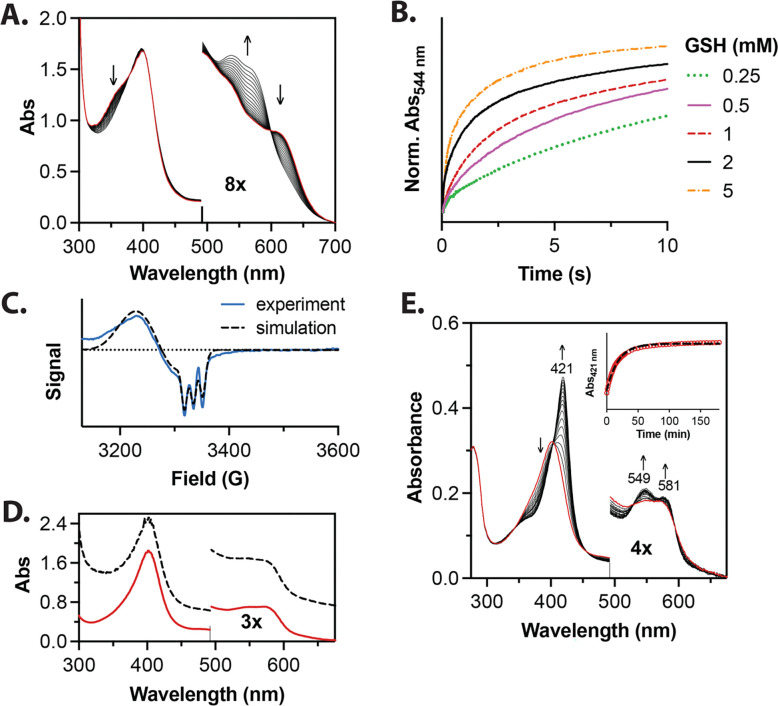
NO-ferroheme formation via glutathione catalyzed reductive nitrosylation of ferric heme taken up into serum albumin. **A)** Spectral changes of 25 μM ferric heme solubilized by 500 μM albumin in PBS reacting with 250 μM NO and 250 μM GSH under anaerobic conditions at 22°C. **B)** As in the MeOH:PBS buffer, rates of ferric heme reductive nitrosylation in albumin increase with GSH concentration. **C)** EPR signature of NO-ferroheme in albumin consistent with a pentacoordinate NO-ferroheme species.The experimental data are shown together with the theoretical simulation used to obtain g-values as described in the methods. **D)** Transfer of NO-ferroheme from membranes to serum albumin. 25 μM ferric heme, 50 μM glutathione, and 50 μM NO were added to red blood cell membrane ghosts under deaerated conditions. Addition of 75 μM serum albumin results in the hashed black line spectrum, which still exhibits typical light scattering due to turbidity from insoluble membranes. To confirm that the NO-ferroheme was transferred from membranes to albumin, the mixture was centrifuged at 30,000g for 2 hours resulting in complete membrane precipitation and pelleting, leaving behind NO-ferroheme in the albumin (red spectrum). **E)** Transfer of NO-ferroheme from albumin to apo-myoglobin in 1:1 ratio of heme to apomyoglobin using a 2 mm cuvette. Nitrosyl-myoglobin is formed given the distinct spectral absorbances at the indicated wavelengths, with isosbestic points from the NO-ferroheme in albumin (red spectrum) over time, indicating direct NO-ferroheme transfer. *Inset*: formation of nitrosyl-myoglobin over time following the Soret formation at 421 nm at 23°C with a half-life of ~12 min under these conditions.

As in the RBC ghost membrane system, glutathione-catalyzed NO-ferroheme formation occurred in the presence of plasma serum albumin. Albumin is an abundant plasma protein that can solubilize a number of hydrophobic compounds. In particular, heme binding to albumin is well-characterized,^58,59^ and heme-nitrosyl species have been observed in human plasma, especially after the inhalation of NO gas.^60^ We observe thiol catalyzed NO-ferroheme formation using serum albumin to solubilize ferric heme and NO-ferroheme product (Figure 3A). Indeed, analogous to both our MeOH:PBS system and red blood cell ghosts system, glutathione accelerates the rate of NO-ferroheme formation and NO in the presence of serum albumin (Figure 3B). Here, we solubilized ferric heme in 500 μM serum albumin, approximating the mammalian plasma serum albumin concentration,^61^ and observed formation of NO-ferroheme upon addition of NO and glutathione (Figure 3A). The rate of NO-ferroheme formation increased as a function of glutathione concentration under pseudo-first order conditions (Figure 3B). Unlike in the MeOH:PBS system, the reaction in the presence of serum albumin exhibits biphasic kinetics under pseudo first-order conditions, discussed further in the supplementary materials (Figure 3B and **Supplementary Figure 2A**). A rhombic EPR spectrum nearly identical to that observed in RBC ghost membranes is observed with *g*-values at 2.085, 2.050, and 2.013 for *g*_max_, *g*_mid_, and *g*_min_, respectively, and a three-line hyperfine splitting centered on *g*_min_ (^NO^A = 47 MHz), consistent with formation of five-coordinate NO-ferroheme species (Figure 3C). Like the MeOH:PBS system, addition of 25 μM ferric heme to 50 uM reduced glutathione and 50 μM NO in 75 μM serum albumin under anaerobic conditions yields roughly one equivalent of NO-ferroheme generated (23.4 ± 4.5 μM), and just under one equivalent of S-nitrosothiol formed (19.4 ± 2.1 μM, **Supplementary Figure 2B**).

### Transfer of NO-ferroheme from membranes to albumin and albumin to apo-myoglobin

As labile heme (and thus potentially NO-ferroheme) can be found in membranes, we qualitatively characterized the transfer of NO-ferroheme formed in our RBC membrane ghosts to serum albumin, the most abundant protein in blood. Ferric heme (25 μM), 50 μM glutathione, and 50 μM NO were added to red blood cell membrane ghosts under anaerobic conditions at 37°C (Figure 3D). This spectrum exhibits substantial light scattering due to the relatively large membrane ghosts. The absorption spectrum after adding 75 μM serum albumin (hashed black line) likewise shows considerable light scattering due to the presence of the membrane ghosts. To confirm that NO-ferroheme was transferred from the membranes to the albumin, the mixture was centrifuged at 30,000 × g for 2 hours resulting in complete membrane precipitation and pelleting, and leaving behind NO-ferroheme solubilized in the albumin (solid red line) accompanied by loss of the membrane-ghost dependent turbidity.

One putative biologically relevant mechanism of action of NO-ferroheme presupposes not only an ability for NO-ferroheme to be ferried from cell membranes and hydrophobic spaces to serum albumin in the plasma for protection and transport, but to then further transfer from albumin to heme binding protein targets. To assess and model such transfer, NO-ferroheme in albumin was added to an equivalent of apo-myoglobin in an anaerobic solution at room temperature (Figure 3E). Though somewhat slow under these conditions with a half-life of roughly 12 min (Figure 3E, *inset*), these data clearly show NO-ferroheme transfer from the NO-ferroheme in albumin (red spectrum) to directly form nitrosyl-myoglobin with signature absorbances of 421, 549, and 581 nm.^62^ Critically, the isosbestic points at 397, 522, and 593 nm indicate direct transfer without formation of any other species or intermediates.

### Stability of NO-ferroheme in oxyhemoglobin

To perform a signaling function, NO-ferroheme must be stable to oxidation and premature NO release under physiological conditions. To measure the stability of NO-ferroheme in albumin under oxygenated conditions, we added aliquots of albumin-solubilized NO-ferroheme to solutions of oxyhemoglobin and followed the formation of methemoglobin spectroscopically (**Supplementary Figure 3**). While free NO is rapidly oxidized by oxyhemoglobin to form nitrate (NO dioxygenation reaction),^13,14,63^ NO complexed into NO-ferroheme should be stable under oxygenated conditions until the NO dissociates away from the heme, as observed with other nitrosyl-hemoproteins such as myoglobin.^64,65^ Consistent with the formation of a stable nitrosyl-heme albumin species, the observed NO dioxygenation by hemoglobin is quite slow for albumin-solubilized NO-ferroheme (8 × 10^−2^ h^−1^ at 23 °C), suggesting slow NO release from NO-ferroheme. These results indicate that nitrosyl-heme in albumin has the potential to form as a relatively stable intravascular species under aerobic conditions and in the presence of oxyhemoglobin.

### In vitro signaling properties of NO-ferroheme with albumin

**Figure 4:**
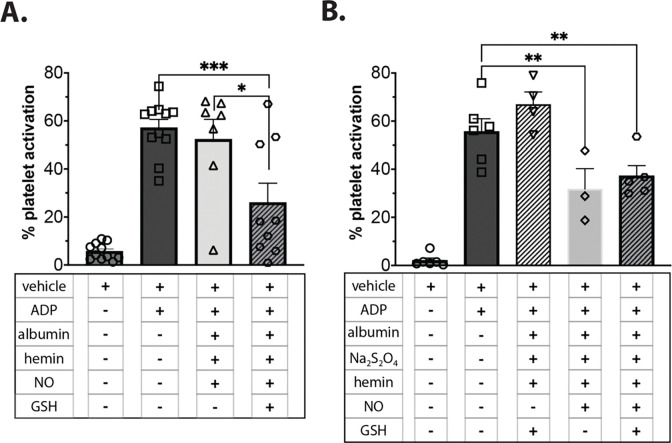
Effects of NO-ferroheme in albumin on platelet activation. Platelet-rich plasma (PRP) was diluted sevenfold with anaerobic PBS. Addition of 2 μM adenosine diphosphate (ADP) to platelets stimulates activation (squares) and was added to all experiments except the vehicle controls (circles) for 10 min. ADP addition was 5 minutes after adding heme albumin samples to PRP. Each symbol within a bar represents data from a different blood donor on a different day. **A)** 2.5 μM heme and 2 μM NO in 7.5 μM albumin were reacted for 35 min resulting in traditional reductive nitrosylation and thus slow and incomplete NO-ferroheme formation. Upon addition to activated platelets, little abrogation of activation was observed (triangles). However, addition of 25 μM glutathione to the reaction results in thiol catalyzed NO-ferroheme formation; significant inhibition of platelet activation was observed upon addition of this sample to platelets (hexagons, p = 0.0002), and significantly more than without GSH in the reaction mixture (triangles, p = 0.0031). **B)** Using the same concentrations as **A**, NO-ferroheme albumin was synthesized using 40 μM sodium dithionite (Na_2_S_2_O_4_). Dithionite did not inhibit platelet activation alone (upside down triangles). However, NO-ferroheme generated in this manner significantly inhibited platelets with and without glutathione present (diamonds, p = 0.004, and hexagons, p = 0.0082, respectively). Statistics were completed using Fisher’s least squared difference test. Platelet experiments were performed at 37 °C

NO is a well-established inhibitor of platelet reactivity via activation of sGC.^66,67^ We hyothesize that generation of NO-ferroheme acts as a physiologically relevant species that shields NO from scavenging until in the proximity of sGC; sGC must then be stimulated, either via NO release or directly binding to apo-sGC,^68–70^ although our experiments incubating NO-ferroheme with albumin and oxyhemoglobin indicate NO release is slow. To establish NO-ferroheme signaling *in vitro*, we isolated platelet-rich plasma (PRP) and triggered platelet activation with 2 μM adenosine diphosphate (ADP, Figure 4).^71^ In the absence of glutathione, addition of 2.5 μM ferric heme and 2 μM NO in 7.5 μM albumin did not significantly inhibit platelet activation (Figure 4A, triangles). However, when solutions containing these same reagents with 25 μM glutathione were added to the activated platelets, significant inhibition was observed (Figure 4A, hexagons), suggesting that NO-ferroheme indeed inhibits platelet activity. Glutathione by itself, ferric heme alone, or heme with albumin or with albumin and glutathione have no effect on activated platelets (**Supplementary Figure 4**).

S-nitrosoglutathione (GSNO) has been shown to inhibit platelets *in vitro.*^72,73^ As GSNO is generated by the reaction, we employed an alternate method to generate NO-ferroheme without GSNO by first reducing hemin with 1.6 fold excess sodium dithionite and then adding an equivalent of NO to simply bind the pre-formed ferrous heme at a final concentration of 2 μM (Figure 4B). Ferrous heme, generated by chemical reduction with dithionite in the absence of NO did not inhibit platelet activation; in fact, it seemed to activate them slightly more than ADP alone (Figure 4B, upside down triangles). However, NO-ferroheme, generated using dithionite and not glutathione, significantly inhibited platelet activation in the presence of absence of glutathione (Figure 4B, diamonds and hexagons). We note that addition of glutathione to the dithionite-prepared NO-ferroheme albumin does not generate GSNO via the 2C assay (data not shown). Taken together, these *in vitro* data suggest that NO-ferroheme in albumin functions as a canonical NO signaling molecule to directly activate sGC in activated platelets.

### In vivo signaling properties of nitrosylated-heme with albumin

**Figure 5:**
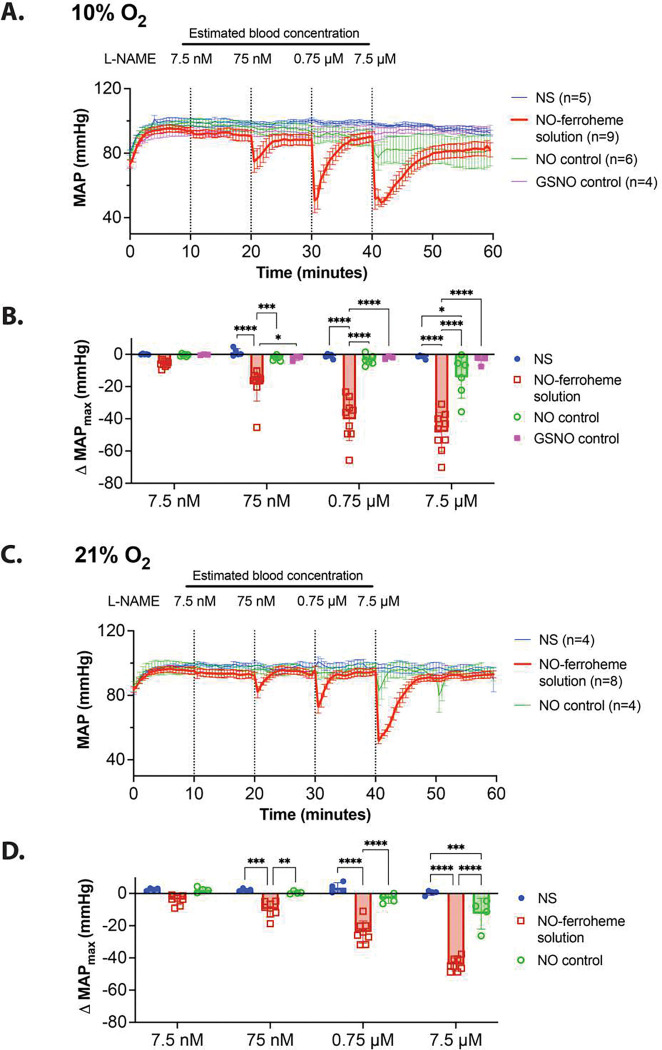
Changes in murine mean arterial pressure (MAP) by NO-ferroheme solubilized by serum albumin prepared via glutathione facilitated reductive nitrosylation. **A)** Under 10% oxygenated breathing conditions and administered L-NAME, both to maximize the sensitivity of changes in MAP, a mouse was administered normal saline (NS, n=5) or one of three regimens: freshly prepared NO-ferroheme solution in 500 μM albumin (300 μM ferric heme, 10 eq. GSH, 2 eq. NO, n=9), a control of dissolved NO in 500 μM albumin (300 μM NO, 10 eq. GSH, n=6), or a control of prepared GSNO in 500 μM albumin (300 μM GSNO, 10 eq. GSH, n=4). NO-ferroheme in albumin or NO or GSNO were administered in doses 10 minutes apart giving estimated blood concentration in the mouse of 7.5 nM, 75 nM, 0.75 μM, and 7.5 μM. **B)** NO-ferroheme in albumin (red open squares) acutely lowered mean arterial pressure in a concentration dependent manner followed by recovery compared to all controls: NS (blue circles), dissolved NO (green open circles), and freshly prepared GSNO (pink squares). Here, the average MAP before an injection was subtracted from the maximum change within 3 minutes of injection (all were an average of five time points) to give the ΔMAP_max_ for each injected species at a given concentration. Statistics were completed using Tukey’s multiple comparisons test; only significant interactions are shown. **C)** The same manner of results as **A**, except under normoxic conditions. **D)** Statistical comparisons between normal saline, NO-ferroheme solution, and the NO control as in **C**, except under normoxic conditions.

To assess whether NO-ferroheme in albumin elicits a vasodilatory response *in vivo,* we synthesized NO-ferroheme solubilized in serum albumin in PBS and performed dose-response experiments in mice, assessing real-time changes in mean arterial blood pressure (MAP) under normoxia and hypoxia (Figure 5). Using male C57BL/6 mice, catheters were inserted into the right carotid artery and jugular vein for monitoring MAP and for dose injections, respectively. We generated fresh NO-ferroheme stock solutions in an anaerobic environment before every experiment, each from 300 μM hemin, 3 mM glutathione, 600 μM NO, and 500 μM bovine serum albumin in PBS. To establish that vasodilation was independent of endogenous endothelial NO synthase (eNOS) derived NO, mice were first treated with L-N^G^-nitroarginine methyl ester (L-NAME) to block basal NO levels from nitric oxide synthases. Four doses of NO-ferroheme were administered (initial dose determined by heme concentration) to result in estimated blood concentrations of 7.5 nM, 75 nM, 0.75 μM, and 7.5 μM, based off a total approximate blood volume of 1.8 mL. Doses were injected intravenously every ten minutes, long enough for the blood pressure to stabilize.

Injections of NO-ferroheme solution in hypoxic animals were characterized by a rapid relaxation followed by a constriction, as monitored by changes in MAP. The MAP almost completely returned to the L-NAME induced baseline after ten minutes (Figure 5A, red MAP trace) when compared to normal saline controls (Figure 5A, blue trace). To quantitatively describe these data, we present the results in terms of the maximum change in MAP (ΔMAP_max_), the average pressures of five sequential timepoints immediately before the injection subtracted from an average of five sequential timepoints at the lowest pressures within three minutes of an injection. The ΔMAP_max_ showed significant vasodilatory effects of NO-ferroheme in albumin (Figure 5B, red bars) versus normal saline controls (Figure 5B, blue bars) for all concentrations (p < 0.0001) except the lowest, 7.5 nM, which still trended toward lower pressures. Moreover, under normoxic conditions, pressures exhibited a similar rapid drop in blood pressure, indicative of vasorelaxation, followed by blood pressure recovery to baseline MAP (Figure 5C, red trace). These changes were also significant versus normal saline at 75 nM, 0.75 μM, and 7.5 μM (p < 0.0002, Figure 5D, red and blue bars).

As this synthesis involves multiple potential vasodilators including NO itself, GSNO, and NO-ferroheme, we needed to define the dominant vasodilating species. We thus compared the vasodilation potency equivalent levels of pure NO in solution and GSNO. We made fresh stocks of an equivalent of NO (300 μM NO, 3 mM glutathione, and 500 μM albumin) and injected mice in 10-minute intervals at the same dosing as the NO-ferroheme albumin injections to yield estimated blood concentrations of 7.5 nM, 75 nM, 0.75 μM, and 7.5 μM dissolved NO in both 10% hypoxia and normoxia (Figures 5A and 5C, green traces). Compared to this dissolved NO control, NO-ferroheme albumin solution triggered significantly more vasorelaxation at all concentrations under hypoxia (Figure 5B, p = 0.0007 for 75 nM, p < 0.0001 for 0.75 μM and 7.5 μM) and normoxia (Figure 5D, p = 0.0015 for 75 nM, p < 0.0001 for 0.75 μM and 7.5 μM). Similarly, using freshly prepared GSNO,^74^ we made fresh controls for the maximum amount of GSNO theoretically generated by this synthesis (300 μM GSNO, 3 mM glutathione, and 500 μM albumin). Following an analogous procedure, GSNO also did not elicit a large change in MAP (Figure 5A, pink trace). Compared to this GSNO control, NO-ferroheme albumin also triggered significantly more vasorelaxation at all concentrations (Figure 5B, p = 0.0116 for 75 nM, p < 0.0001 for 0.75 μM and 7.5 μM). Compared to normal saline, the NO and GSNO controls exhibited no significant changes in MAP, except the highest dose of dissolved NO (7.5 μM: hypoxia, p = 0.0397; normoxia, p = 0.0004).

### Identification of NO-ferroheme as the vasodilating species

Though the use of glutathione presents a physiological and kinetically viable way in which labile ferric heme may be converted into NO-ferroheme, generating the solution *in vitro* results in a mixture of vasodilating species, including GSNO. As described during our platelet experiments, we synthesized the NO-ferroheme albumin using sodium dithionite to avoid GSNO formation. To generate pre-formed ferroheme, 300 μM hemin and 330 μM sodium dithionite were mixed in a 500 μM solution of serum albumin in PBS under anaerobic conditions. 300 μM of NO was then added, resulting in rapid formation of NO-ferroheme in albumin as confirmed by UV-Vis spectroscopy.

**Figure 6:**
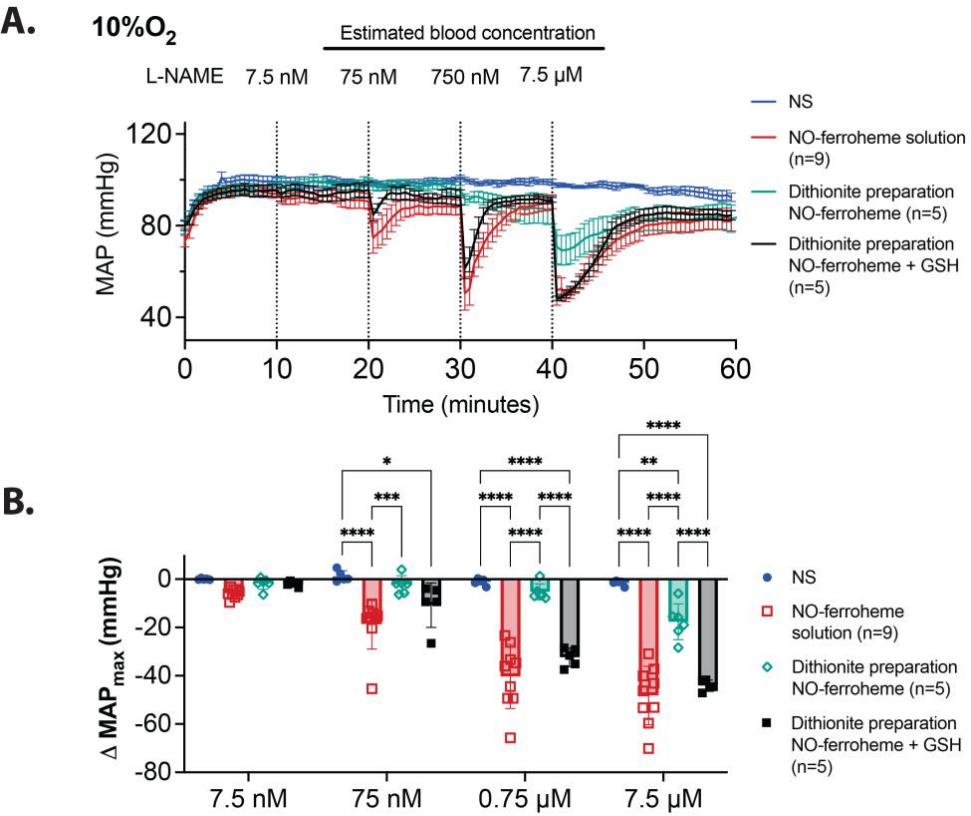
Changes in murine mean arterial pressure (MAP) by NO-ferroheme prepared via sodium dithionite reduction and addition of NO solubilized by serum albumin. **A)** Akin to Figure 5A, under 10% oxygenated breathing conditions and administered L-NAME, a mouse was administered NO-ferroheme albumin prepared by hemin reduction by 10% excess sodium dithionite (teal line, n=5). By itself, this did not have the same vasodilatory activity observed in NO-ferroheme albumin solution prepared via glutathione catalyzed reductive nitrosylation (red line, n=9). However, addition of the 3 mM glutathione to this solution restored such activity (black line, n=5). These regimens were administered in doses 10 minutes apart giving estimated blood concentration in the mouse of 7.5 nM, 75 nM, 0.75 μM, and 7.5 μM of each preparation. **B)** ΔMAP_max_ for each injected species described in **A** at a given concentration. Statistics were completed using Tukey’s multiple comparisons test; only significant interactions are shown.

Intravenous infusion of this glutathione-free preparation of NO-ferroheme resulted in more modest vasodilation at the highest tested concentration (Figure 6, teal trace and open diamonds; p = 0.0023 at 7.5 μM vs normal saline). This response, which is blunted in comparison to injections of NO-ferroheme albumin solution generated via glutathione-catalyzed reductive nitrosylation (Figure 5 and Figure 6, red traces), suggests that glutathione plays a role in mediating the NO-ferroheme-dependent vasodilatory response. As GSNO exhibited no effect on the MAP under these conditions (Figure 5A and 5B), the formulation difference must involve the thiol in another mechanism. To evaluate this possibility, reduced glutathione was added to the dithionite preparation of NO-ferroheme to test the effects of thiol addition without GSNO formation. The addition of reduced glutathione significantly restored the potent vasodilation (Figure 6, black trace and closed squares). No significant differences at any concentration were observed between this dithionite preparation with glutathione added and the original preparation of NO-ferroheme (Figure 6B, black closed squares vs red open squares, respectively). We confirmed that S-nitrosation does not occur in this preparation, and thus there is no GSNO present in the dithionite preparation of NO-ferroheme. These findings suggest that GSH effects NO-ferroheme albumin delivery: it may affect their relative equilibrium, maintain a stable redox environment, or perhaps result in mixed disulfide formation resulting in a change in albumin protein dynamics.

## Discussion

The presented studies identify a kinetically fast chemical reaction pathway to form a novel NO signaling molecule that is stable in the presence of oxyhemoglobin. Under standard reductive nitrosylation conditions, labile ferric heme slowly forms nitrosyl ferrous heme with a half-life of about 340 s with 100-fold excess NO. However, addition of a small amount of a reduced thiol, such as glutathione, greatly accelerates the formation of NO-ferroheme, lowering the half-life of formation to 1 s even with tenfold less NO under otherwise analogous conditions. The NO-ferroheme species is stabilized when solubilized by cell membrane and when bound to albumin, has a long half-life even in the presence of excess oxyhemoglobin, and exhibits potent canonical NO signaling effects of platelet activation and vasodilation.

**Scheme 1:**
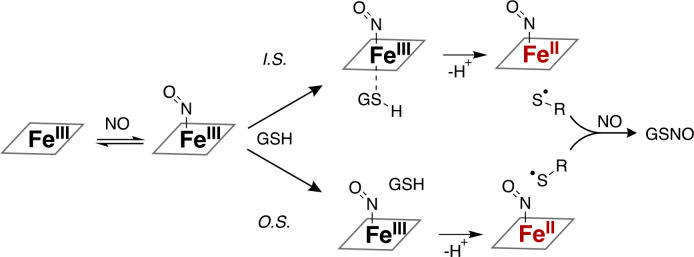


Mechanistically, glutathione serves as a reducing partner for the ferric heme, and accelerates the formation of the ferrous nitrosyl.^26^ However, glutathione does not directly reduce the labile ferric heme,^43^ suggesting that NO association is required for glutathione-catalyzed heme reduction. Kinetic fits under pseudo-first order conditions (where NO and glutathione are at least in fivefold excess compared to heme) yield single exponentials, and the spectral changes exhibit clean isosbestic points with no intermediate species observed. Sub-stoichiometric equivalents of thiol also accelerate the reaction (data not shown). Under biological conditions, thiols oxidized in the process of generating NO-ferroheme would be rapidly re-reduced by cellular reductases, making the process catalytic. Together these observations suggest a sequential mechanism where NO binds ferriheme, forming a ferric nitrosyl that is subsequently reduced by glutathione (Scheme 1), though the possibility that glutathione binds first cannot be excluded. Such a mechanism has been considered before,^26,75,76^ however, thiyl radical formation was never confirmed and rates not quantified. In our study, one electron reduction of the ferric heme-NO by glutathione and concomitant formation of a transient thiyl radical was confirmed using excess of the radical spin-trap DMPO. Taken together, an electron transfer mechanism is implicated (Eqs. 4 and 5) rather than generation of an electrophilic nitrosonium that is typical of reductive nitrosylation (Eqs. 3 and 2), though the one-electron reduction may proceed via either an inner sphere (I.S.) or an outer sphere (O.S.) reaction (Scheme 1).

The formation of NO-ferroheme in red cell membranes is consistent with the hydrophobic nature of labile heme. One would expect that if NO-ferroheme was formed in red blood cells, it would occur on or in lipophilic compartments such the plasma membrane. Further, red blood cells are known to release heme into plasma,^77^ though the mechanisms of heme cellular export are not as well established. However, a recently discovered heme transport protein, feline leukemia virus subgroup C receptor 1 isoform a (FLVCR1a), has been found in the plasma membrane of red blood cells.^1,39,77,78^ As the biochemical mechanism is not fully established,^1^ FLVCR1a, or other yet to be established transporters, may provide a means for cellular export of NO-ferroheme into the plasma, where it can be picked up by serum albumin as we have observed *in vitro*; albumin binds significantly more labile heme overall than the heme-chelating specific plasma protein, hemopexin.^79,80^ Another known heme exporter found in erythrocytes and other cells is ATP-binding cassette super-family G member 2 (ABCG2). This transporter is suspected to export and transfer heme directly to serum albumin,^2,42,81,82^ and therefore may act as a functional transporter for NO-ferroheme as well. At any given time there is 1.5 – 50 μM heme-albumin found in human circulation.^59^ Some of this may be NO-ferroheme, and specifically detecting NO-ferroheme in the vasculature requires further study. Alternatively, NO-ferroheme may also be readily formed in albumin itself in the presence of glutathione and NO from ferric heme. A well-defined heme binding pocket has been characterized in serum albumin, which axially coordinates heme via Tyr161, though heme binds non-specifically in other lipophilic areas as well.^58,83,84^ However, the EPR signal of NO-ferroheme in albumin in our studies suggests a single five-coordinate iron site with only axial NO bound.^55,56^ Combined with the observed transfer of NO-ferroheme from albumin to apomyoglobin to directly generate nitrosyl-myoglobin, these results suggest that NO-ferroheme can be generated and shuttled, both from membranes to albumin and albumin to heme-free hemoproteins.

The observed vasodilation and platelet inhibition suggest NO derived from NO-ferroheme, or more probably NO-ferroheme itself, directly binds and activates sGC in smooth muscle and platelets, respectively. We know that NO-ferroheme in albumin is protected against NO scavenging via the dioxygenation reaction with oxyhemoglobin, increasing the effective lifetime of NO in a physiological environment like blood by several orders of magnitude. In fact, the NO *k*_*off*_ from NO-ferroheme in the albumin binding pocket has been reported to be 1.4 × 10^−4^ s^−1^,^85,86^ and yet we observe a rapid and robust vasodilatory response, conceivably through shuttling the NO-ferroheme itself. In our mouse model experiments, intravenous infusion of albumin-solubilized NO-ferroheme elicited a strong global vasodilatory effect at the low-to-mid nanomolar range in circulation. The response under comparable hypoxic conditions was more potent than nitrite – a known vasodilator and critical stable endocrine reserve of NO.^87,88^ Injection of an equivalent of dissolved NO under otherwise analogous conditions exhibited little response related to red blood cell hemoglobin scavenging. As an equivalent of GSNO should be produced in our synthesis of NO-ferroheme via our determined mechanism, barring other protein/small molecule reactions to consume the generated thiyl radical, we tested purified GSNO in this concentration regime. Similar to dissolved NO, GSNO under these conditions had no effect, consistent with prior published data that GSNO does not significantly effect mean arterial pressure in rodents at concentrations less than 300 μM.^89^

Interestingly, NO-ferroheme produced without thiol using sodium dithionite to reduce the heme showed a weaker vasorelaxation response at all tested concentrations *in vivo*. This observation contrasts with our experiments *in vitro* inhibiting platelet activation, where both the thiol catalyzed NO-ferroheme and the dithionite-generated NO-ferroheme without glutathione were equally effective inhibitors. However, addition of the thiol after NO-ferroheme formation via dithionite rescued the *in vivo* vasodilatory effect. Taken together, these data suggest that addition of thiol either stabilizes the NO-ferroheme albumin or facilitates transport of albumin to smooth muscle. The thiol may affect the NO-ferroheme/albumin equilibrium by altering the redox environment or may otherwise trigger mixed disulfide formation effecting protein dynamics and perhaps cellular import. Alternatively, the possibility cannot be excluded that NO-ferroheme with GSH axially ligated is triggering vasorelaxation, although we observe no spectroscopic evidence of such a species *in vitro*.

A possible candidate for import of NO-ferroheme bound albumin is the transferrin receptor (CD71), which is known to specifically endocytose heme-albumin into cells and use it as an iron/heme source in several cell types, promoting proliferation in the absence of transferrin.^79^ Moreover, CD71 is expressed on the surface of many cell types, including vascular smooth muscle cells, endothelial cells, and cardiomyocytes.^90,91^ Albumin is also known to cross endothelial cells in a processes called transcytosis via albumin activation of endothelial surface glycoprotein gp60, providing another potential route for albumin-solubilized NO-ferroheme to reach smooth muscles cells and other tissues.^92^ NO could be released from NO-ferroheme bound albumin at the primary binding site once imported; this site is allosterically controlled by binding of other macrocycles at ‘Sudlow’s sites,’ specifically pushing His146 to replace the NO to make a hexacoordinate heme with Tyr161.^59,85^ However, as originally hinted at by the Ignarro group, and consistent with observed NO-ferroheme transfer from membrane to albumin and subsequently from albumin to apo-myoglobin, NO-ferroheme itself, once released in the cytosol, may directly activate heme-deficient apo-sGC.^28,30^ The details of NO/NO-ferroheme release of release to stimulate vasorelaxation are the subject of our future studies.

## Conclusions

In this manuscript, we have shown previously unexplored chemistry in which physiologically abundant reduced glutathione facilitates the rapid reductive nitrosylation of ferric heme, resulting in the formation of NO-ferroheme and a thiyl radical, which in the presence of excess NO yields a S-nitrosothiol. We suspect NO-ferroheme represents a novel signaling molecule that helps shield NO from autocapture and dioxygenation on the way to signaling sGC. Indeed, even low-to-mid nanomolar concentrations in circulation of NO-ferroheme in serum albumin engenders a strong vasodilatory response. While numerous questions remain regarding transport as well as cellular import and export, formation of NO-ferroheme may provide an answer in the NO signaling field as to how relatively ephemeral NO survives highly reactive species such as oxyhemoglobin in the red cell and oxymyoglobin in cardiac and skeletal muscle cells.

## Materials and Methods

### Materials and solution preparation

All chemicals were obtained from Sigma Aldrich (St. Louis, MO) unless otherwise noted. The nitric oxide donor ProliNONOate was purchased from Cayman Chemical (Ann Arbor, MI), and all stock solutions were prepared anaerobically in NaOH fresh before use. Nitric oxide gas was obtained from Matheson Tri-Gas, Inc. (Irving, TX) and was bubbled through 1 M NaOH solution before use to remove NO_2_ (g) impurities. Methanol solvent (HPLC grade) was obtained from Fischer Scientific (Waltham, MA). L-glutathione was obtained from Alexis Biochemicals (San Diego, CA). S-nitrosoglutathione was both purchased and made as previously described.^74^ Buffers were prepared with 0.1 to 0.5 mM EDTA where appropriate to eliminate confounding effects of free metal ions on oxidation of thiols and S-nitrosothiol degradation. All experiments were performed in 10 mM phosphate buffer saline, pH 7.4, unless otherwise stated and pH measurements were performed with a Fisherbrand Accumet pH meter equipped with a Hamilton MiniTrode. FITC labeled PAC-1 and Per-CP labeled CD61 antibodies were purchased from BD Biosciences. All animal studies were performed using protocols approved by the Institutional Animal Care and Use Committee at the University of Pittsburgh and in accordance with National Institutes of Health guidelines (Protocol # 21110109). Human blood and plasma were collected using protocols approved by the Institutional Review Board at Atrium Health Wake Forest Baptist, Wake Forest Medical School (Protocol # BG02-168). Both male and female volunteers were recruited, and information was not gathered with respect to gender. Healthy volunteers were recruited by word of mouth between the ages of 17 and 60, but no limitations were made on recruiting older adults. Informed consent was given. Hemoglobin was prepared by washing whole blood in normal saline and osmotic lysis of the red blood cells. Red cell membranes were sedimented at 30,000 g and the hemoglobin was then extensively dialyzed against deionized water and subsequently phosphate buffered saline. Figures were generated and statistics performed using GraphPad Prism v 9.4 and Microsoft Excel.

### UV-Visible Spectroscopy and Kinetics

All individual spectra and pseudo-first order kinetics were performed using a thermostatted (23/37°C ± 0.2) Cary 50, Cary 100, or HP8453 UV-Visible spectrophotometer (Agilent Technologies) for reactions ferric heme reactions with nitric oxide (NO) without glutathione in PBS and MeOH/PBS buffer (1 part PBS in 5 parts methanol) unless otherwise noted. For pseudo-first order reactions with fast observed rates such as the ferric heme reaction with NO and reduced glutathione, a thermostatted SX-20 stopped-flow instrumented fitted with a direct mount photodiode array (Applied Photophysics, Ltd.) in an anaerobic chamber was used. Samples were prepared either in a glove box under nitrogen atmosphere or using a Schlenk line under argon atmosphere with oxygen-free buffers and solvents.

### Preparation of NO-ferroheme in methanol and PBS solution

Hemin stock (5 mM) was prepared regularly in 20 mN anaerobic NaOH solution, sealed in a septum capped vial. The concentration of hemin was precisely determined by absorption spectroscopy using the extinction coefficient at 385 nm of 58.4 mM^−1^cm^−1^.^93^ Reduced glutathione stocks (25 – 100 mM) were made regularly in deaerated PBS and sealed in septum capped vials. Methanol was deaerated separated before mixing in a 5:1 ratio with deaerated PBS in an anaerobic chamber to make MeOH:PBS buffer for spectroscopy experiments. The precise concentration of NO from bubbling or from ProliNONOate was determined either by titrating with excess oxyhemoglobin to a ~1:2 molar ratio and calculating the amount of methemoglobin produced by fitting absorption spectra to normalized basis spectra or using an ozone-based chemiluminescent analyzer. Stock concentrations of ProliNONOate were also verified through absorption spectroscopy, using the extinction coefficient at 248 nm of 8.4 mM^−1^cm^−1^.^94^ A sample stopped-flow experiment follows: 25 μM hemin and 250 μM hemin were loaded into one syringe of the stopped-flow. To the other syringe, a range of NO concentrations were loaded, between 125 μM and 2.5 mM. Note that these concentrations are double the observed concentrations, as upon mixing in the stoppled flow, all concentrations are halved. For radical trapping experiments, the above reaction conditions were replicated plus the addition of 50 mM DMPO in deaerated MeOH:PBS solution.

### Chemiluminescent assays of NO-ferroheme and S-nitrosothiols

A Zysense (formerly Sievers) Nitric Oxide Analyzer 280i or an ECO PHYSICS nCLD 88 liquid NO system was used to detect NO congeners. Measurements of nitrosothiols was made using Cu/Cys assay (2C assay)^48^ in a chemiluminescent nitric oxide analyzer by injecting each sample into the NOA purge vessel containing the Cu/Cys reagent at 50°C. The Cu/Cys reagent was prepared by adding 100 mL each of 1 mg/mL copper chloride and 100 mM L-Cysteine in 10 mL of Sodium Phosphate buffer pH 6.5 at 50°C. NO-ferroheme was measured by injecting an amount each sample into the NOA purge vessel containing 50 mM potassium ferricyanide in PBS at 25 or 37°C. See Results section for specific amounts. Samples were protected from light by aluminum foil.

### Preparation of NO-ferroheme in albumin solution (heme-nitrosyl albumin)

Stocks of hemin, glutathione, and NO were prepared as described above. An albumin stock (0.5 – 1 mM) was made in phosphate buffered saline (PBS) and the concentration verified by absorption spectroscopy using an extinction coefficient of 43.8 mM^−1^cm^−1^ at 280 nm. Stock solutions were used to produce mixtures on a Schlenk line and transferred using Hamilton syringes with concentrations given in the results section. Solutions were made directly or indirectly and transferred, with no differences between products of each preparation. The direct method involved adding ferric heme solution and glutathione directly to albumin in an argon or nitrogen filled septum-sealed vial, followed by NO addition. Within seconds, the solution changes from a brownish color to a deep red color, with no trace of turbidity. Solutions were used after five minutes. The indirect method involved NO addition to heme and GSH in PBS before then adding albumin after five minutes. The NO-ferroheme was then incubated with albumin for thirty minutes.

### Preparation of red blood cell membrane ghosts

Red cells were separated from the whole blood by sedimentation via centrifugation at 1000 g for 10 minutes. These packed red cells were then hemolyzed using 5 mM phosphate buffer, pH 8 (4.674 mM Na_2_HPO_4_ + 0.326 mM NaH_2_PO_4_) in the volume ratio of 1:40 for an hour. The mixture was spun at 35,000 g for 30 minutes at 20°C and washed with the buffer repeatedly until the white ghosts were produced. The absence of hemoglobin was confirmed by taking the absorption of the ghosts in the range of 300 – 700 nm.

### EPR spectroscopy

Electron paramagnetic resonance (EPR) spectra were recorded on a Bruker EMX spectrometer operating at 9.4 GHz, 5-G modulation, 10 milliwatt power, 328-ms time constant, and 164-s scan over 600 G at 110 K or 10 K as described previously.^95^ Two scans were taken and averaged for each sample. Concentrations were calculated after double integration using heme-nitrosyl basis spectra with known concentrations. For power saturation measurements, spectra were taken with 0.1, 1, and 10 mW power (33, 23, 13 decibels). For radical trap experiments, the *g*-values and hyperfine tensors for each EPR spectrum were extracted from simulations performed using EasySpin (v5.2.23).^96^

### NO-ferroheme transfer from membranes to albumin

Heme (25 μM) 50 μM NO, and 50 μM GSH were reacted for 30 minutes in the presence of red cell membrane ghosts under anaerobic conditions. The sample was incubated for another 30 minutes at 37°C after adding 75 μM albumin. After the incubation, the sample was scanned for absorption in the Cary 50 UV-Vis spectrometer. The sample was subsequently spun at 30,000 g for two hours in a Sorvall^R^ RC-5B ultracentrifuge and the absorption spectrum of the supernatant was measured.

### NO-ferroheme transfer from albumin to apo-myoglobin

NO-ferroheme in albumin was made using 25 μM Heme and 50 μM NO mixed for five minutes under anaerobic conditions, in pH 7.4 PBS buffer, in the presence of GSH (50, 125, 250 μM) before adding albumin. The NO-ferroheme was incubated with 25 mM albumin for 30 minutes before mixing with 30 μM apomyoglobin under anaerobic conditions. The kinetics were monitored for 330 minutes by UV-Vis absorption spectroscopy using 2mm quartz cuvettes. No GSH dependence on the kinetics was observed.

### Platelet activation

Samples were prepared using heme (25 μM), NO (20 μM), albumin (75 μM) with and without GSH (250 μM) under normoxic or anoxic conditions. Fresh blood was drawn into sodium citrate tubes with the first tube being discarded and platelet rich plasma (PRP) collected after sedimentation of the red blood cells. Platelet activation was measured as described previously using platelet agonist ADP.^97^ First, PRP was diluted 1:7 with oxygenated or deoxygenated PBS buffer, then albumin samples were mixed with this diluted PRP in 1:10 ratio. After 5 minutes of incubation at 37°C, 2 μM ADP was added to all test samples except negative vehicle controls, followed by 10 additional minutes incubation. 20 μL of each sample was subsequently added to PAC-1 FITC (labels activated platelets) and CD61 (labels all platelets) antibodies and incubated at room temperature in the dark for 20 minutes and then fixed in 1% buffered formaldehyde. Platelets were sorted using a BD FACSCalibur Analyzer. Gating strategy was based on side and forward scattering compared to red blood cells and then also for activated platelets. Platelets were labelled along the x-axis and activation on the y-axis. Activation was taken as the upper right quadrant divided by the sum of the two right quadrants (**Supplementary Figure 5**).

### In vivo vasodilation experiments

Male C57BL/6 mice (the Jackson Laboratory), age between 12 to 14 weeks, were anesthetized by isoflurane (2.5%). Body temperature was maintained at 37°C by a heating pad connected to heat pump (Androit). Catheters were implanted for carotid artery and jugular vein. Arterial blood pressure was continuously recorded through a blood pressure transducer (MLT699, ADInstrument) connected to an 8-channel Powerlab (ADInstrument). A tracheal tube was introduced and connected to a rodent ventilator (Model 849, MidiVent). Upon completion, in some animals, the fraction of inspiratory O_2_ was decreased from 21% to 10% to induce hypoxia. 75μL of L-NAME was administered intravenously in a bolus at a dose of 10 mg/kg bodyweight. 10 minutes after L-NAME injection, animals were injected with 50 μL of NO-ferroheme or normal saline (NS) for 4 times at 10-minute intervals. An NO-ferroheme in albumin stock was prepared via a concentrated version of the direct method as described above: 300 μM ferric heme was mixed anaerobically on a Schlenk line with 3 mM GSH and 600 μM NO in 500 μM albumin using air-tight Hamilton syringes. This stock was diluted further in air with albumin solution before injection by 1000-, 100-, and 10-fold. Mean arterial pressure (MAP) values were obtained by processed the recorded arterial pressure data with LabChart 7.3.8 (ADInstruments).

## Data Availability

The datasets generated during and/or analyzed during the current study are available from the corresponding authors on reasonable request.
